# The Relationship Between Cardio-Ankle Vascular Index and Left Atrial Phasic Function in Hypertensive Patients With Preserved Ejection Fraction

**DOI:** 10.3389/fmedt.2021.724089

**Published:** 2021-08-31

**Authors:** Tsuyoshi Tabata, Kazuhiro Shimizu, Yukihiro Morinaga, Naoaki Tanji, Ruiko Yoshida, Masahiro Iwakawa, Hajime Kiyokawa, Nobuo Takada

**Affiliations:** ^1^Department of Clinical Functional Physiology, Toho University Sakura Medical Center, Chiba, Japan; ^2^Department of Internal Medicine, Toho University Sakura Medical Center, Chiba, Japan

**Keywords:** arterial stiffness, cardio-ankle vascular index, hypertension, left atrial phasic function, speckle-tracking echocardiography

## Abstract

**Background:** To investigate the relationship between arterial stiffness, reflected by cardio-ankle vascular index (CAVI) value, and left atrial (LA) phasic function in hypertensive patients with preserved left ventricular ejection fraction (LVEF).

**Methods:** We retrospectively studied 165 consecutive patients (mean age, 66.5 ± 11.7 years) diagnosed with hypertension with preserved LVEF who had undergone CAVI measurement and echocardiography on the same day. The latter included speckle-tracking echocardiography to assess LA phasic function (reservoir, conduit, and pump strain) and left ventricular global longitudinal strain (LVGLS).

**Results:** The results of univariate analysis showed CAVI value to be correlated with LA reservoir strain and LA conduit strain (*r* = −0.387 and −0.448, respectively; both *P* < 0.0001). The results of multiple linear regression analysis showed CAVI value to be independently related to age (β = 0.241, *P* = 0.002) and LA conduit strain (β = −0.386, *P* = 0.021) but not LV mass index, LA volume index, or LV systolic function (including LVGLS).

**Conclusion:** In hypertensive patients with preserved LVEF, increased CAVI value appears to be independently associated with impaired LA phasic function (particularly LA conduit function) before LA and LV remodeling. CAVI determination to assess arterial stiffness may be useful in the early detection of interactions between cardiovascular abnormalities in hypertensive patients.

## Introduction

Arterial stiffness increases the risk of cardiovascular morbidity and mortality in patients with hypertension. It impairs blood flow from left atrium to left ventricle, thus increasing left atrial (LA) pressure and preload. As the left atrium tries to compensate for the resulting physiological effects, it becomes dilated and functionally impaired. These changes in LA size and function progress in proportion to the degree of left ventricular (LV) dysfunction (1).

Arteriosclerosis is a generic term for arterial lesions characterized by thickening, hardening, and remodeling of the arterial wall. Under this term, three lesions are listed: atherosclerosis, arteriolosclerosis, and Mönckeberg's sclerosis with calcification. The cardio-ankle vascular index (CAVI) is now widely used to evaluate arterial stiffness independently of blood pressure. It can be used to predict cardiovascular events and thereby help manage cardiovascular risk (2, 3).

LA makes three performances (reservoir during ventricular systole, conduit during early ventricular diastole, and pump during late ventricular diastole). Early detection of abnormalities in LA dysfunction is clinically important. The recent development of two-dimensional speckle-tracking echocardiography (2DSTE) has enabled subtle changes in LA reservoir, conduit, and pump function to be detected and quantitated (4). Some investigations have pointed out that 2DSTE enables the detection of LA dysfunction in hypertensive and diabetic patients before the manifestation of LA enlargement (5).

The relationship between arterial stiffness measured by CAVI and LA phasic function was also reported previously by Yoshida et al. (6). The LA function consists of three elements: reservoir, conduit, and pump function. To understand more deeply about increased arterial stiffness and these LA functions, we investigated the relationship between arterial stiffness and LA functions in mild hypertensive patients with preserved left ventricular ejection fraction (LVEF) at the same day.

## Materials and Methods

### Study Patients

In this retrospective observational study, we used data from 165 consecutive patients with a diagnosis of hypertension and with preserved LVEF who underwent CAVI measurement and echocardiography on the same day at Toho University Sakura Medical Center (Chiba, Japan) between 2015 and 2018. Exclusion criteria included LVEF <50%, acute myocardial infarction, old myocardial infarction, cardiomyopathy, acute myocarditis, open-heart surgery, non-sinus rhythm, atherosclerosis obliterans (ankle-brachial index, <0.9), and significant valvular disease (moderate or severe mitral and/or aortic valvular disease).

Patients were defined as having hypertension if they had systolic blood pressure ≥140 mmHg and/or diastolic blood pressure ≥90 mmHg or were receiving antihypertensive treatment. The presence of the following concomitant diseases, considered cardiovascular risk factors, was recorded: diabetes mellitus (defined as glycated hemoglobin ≥6.5% [NGSP-standardized value] or requiring antidiabetic treatment) and dyslipidemia (defined as low-density lipoprotein cholesterol concentration ≥140 mg/dL, high-density lipoprotein cholesterol concentration <40 mg/dL, triglyceride concentration ≥150 mg/dL, or requiring antihyperlipidemic treatment).

### Measurement Cardio-Ankle Vascular Index

All CAVI values were determined from measurements obtained with a vascular screening system (VaSera1500; Fukuda Denshi Co., Ltd., Tokyo, Japan) from patients examined in a quiet room in which a constant temperature was maintained. The method used was as described previously (2, 3). Briefly, cuffs were applied to the bilateral upper arms and ankles, with the patient supine and his or her head held in the midline position. After the patient had been allowed to rest for 10 min, low cuff pressure (30–50 mmHg) was used to enable detection of both brachial and ankle pulse waves with minimal effect on hemodynamics. Blood pressure was subsequently measured.

The CAVI value was determined with the following equation, derived from the Bramwell–Hill equation:

CAVI value = a {(2*p*/Δ*P*) × In (*P*_s_/*P*_d_) *PWV*^2^} + b,

in which *P*_s_ and *P*_d_ are systolic and diastolic blood pressure, respectively; *PWV* is pulse-wave velocity from the origin of the aorta to the tibial artery-femoral artery junction; Δ*P* is the difference between systolic and diastolic blood pressure (i.e., *P*_s_ – *P*_d_); *p* is blood density; and a and b are constants. CAVI value was adjusted for blood pressure based on the stiffness parameter β. We used CAVI values determined from measurements obtained from the right ankle in this study.

**Figure 1:**
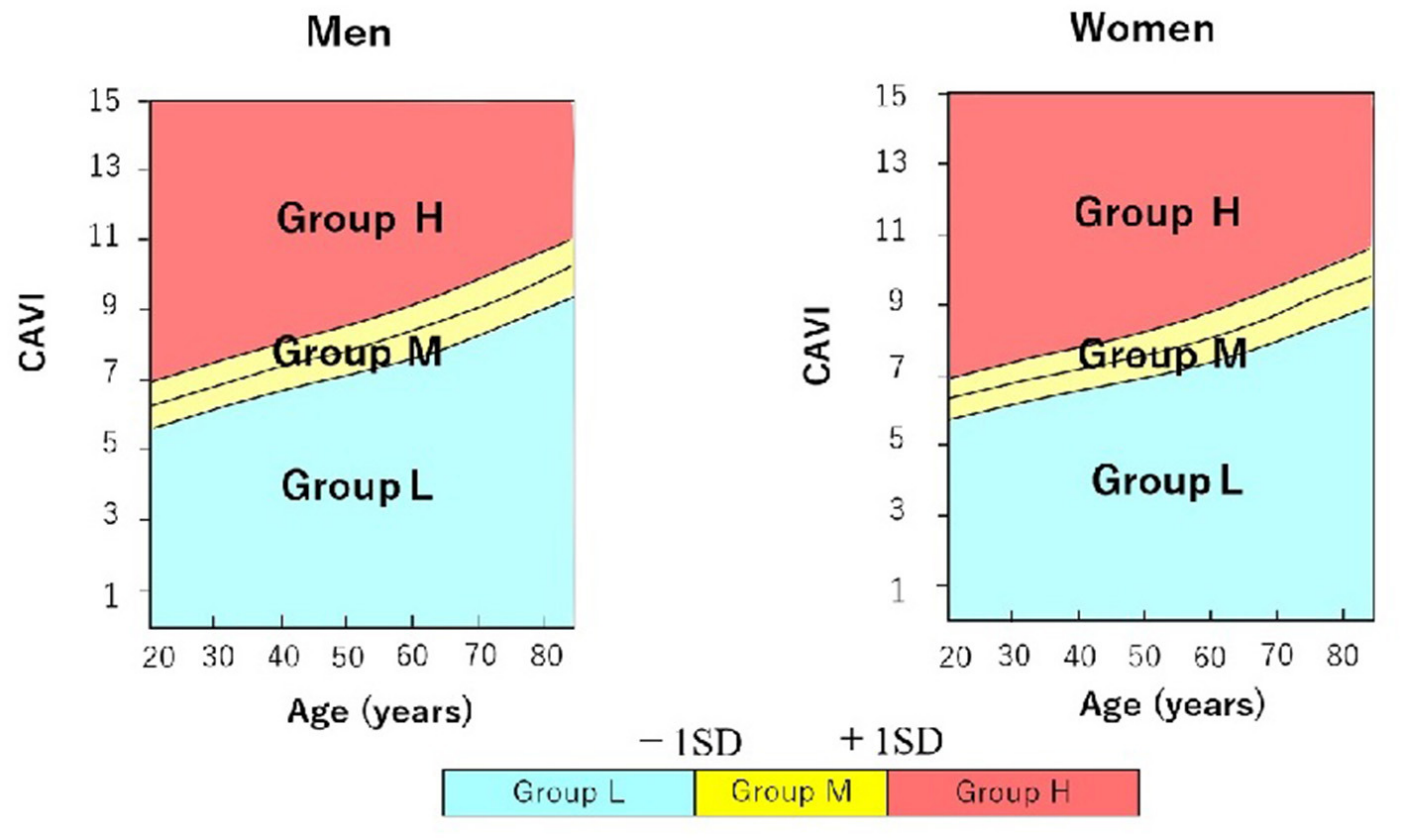
Stratification of male and female hypertensive patients with preserved ejection fraction into 3 groups according to cardio-ankle vascular index (CAVI) value relative to mean CAVI value for age. Group L (*n* = 34; 23 men, 11 women) comprised patients with low CAVI value(≥1 standard deviation, SD, lower than mean for age); group M (*n* = 94; 73 men, 21 women) comprised patients with medium CAVI value (± 1 SD of mean for age); and group H (*n* = 37; 23 men, 14 women) comprised patients with high CAVI value (≥1 SD higher than mean for age). CAVI, cardio-ankle vascular index; SD, standard deviation.

We used the reference values provided by a large-scale physical examination of the Japanese healthy population. Values of CAVI increase with age and are higher in men than in women regardless of age (7). Therefore, to exclude the influence of age and sex, we stratified patients into 3 groups according to CAVI value relative to mean CAVI value for age: group L, patients with low CAVI value (≥1 standard deviation, SD, lower than mean for age); group M, patients with medium CAVI value ( ± 1 SD of mean for age); and group H, patients with high CAVI value (≥1 SD higher than mean for age) (Figure 1).

### Echocardiographic Examination

#### Two-Dimensional Echocardiography

All echocardiographic examinations were carried out using a commercially available system (Vivid7, E9, S5, and S6; GE Healthcare, Boston, MA, USA) in accordance with the standardized protocol. The dimensions of the cardiac chambers were measured in the standard manner (8). The biplane Simpson's method was used to determine LVEF.

Left ventricular mass was calculated using the following validated formula:

LV mass = 0.8 (1.04 [(*IVS* + *LVEDD* + *PWT*)^3^ – *LVEDD*^3^]) + 0.6,

in which *IVS* is LV end-diastolic interventricular septal thickness, *LVEDD* is LV end-diastolic diameter, and *PWT* is LV end-diastolic posterior wall thickness (9). The Simpson's method was used to calculate LA volume from measurements obtained in the apical 4-chamber and 2-chamber views (8).

The LV mass and LA volume were then indexed to body mass area. LV diastolic function was assessed in accordance with current guidelines (10). The pulsed Doppler method was used to determine transmitral flow velocity in the apical 4-chamber view. Peak early diastolic velocity (E wave), peak atrial systolic velocity (A wave), and *E*/*A* ratio were determined from the transmitral flow-velocity pattern (*E*, peak early diastolic velocity of transmitral flow; *A*, peak atrial systolic velocity of transmitral flow). Pulsed tissue Doppler echocardiography was used to determine mitral annular motion velocity at the LV lateral wall and septal wall sites in the apical 4-chamber view. Peak early diastolic motion velocity (*e*') and *E*/*e*' ratio were determined, with *e*' being the average of the septal and lateral annulus.

#### Speckle-Tracking Echocardiography

Left atrial strain was quantitated offline, using the EchoPAC PC system, version 113 (General Electric Healthcare, Chicago, IL, USA). The software detects borders semi-automatically, and tracks the LA borders throughout the entire cardiac cycle. Cases of inaccurate endocardial detection were corrected manually. LA strain was determined as the average of values obtained for six LA segments in the apical 4-chamber and 2-chamber views. The reference for zero strain was set at LV end-diastole (R-R triggering), in accordance with current recommendations (11).

**Figure 2:**
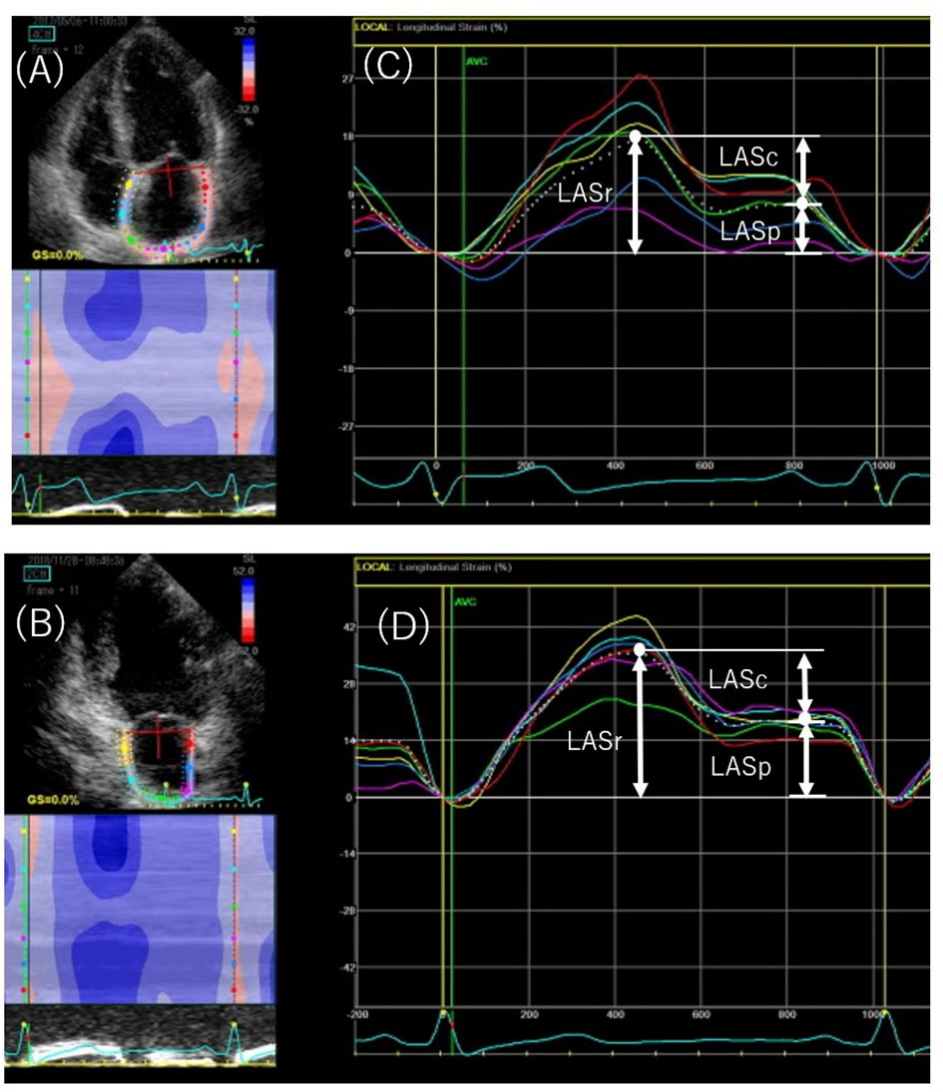
Speckled-tracking echocardiography to assess left atrial (LA) phasic strain. Endocardial region of interest in the apical 4-chamber **(A)** and 2-chamber **(B)** views. The peak positive longitudinal strain (LASr) corresponds to LA reservoir function, and the strain during early and late diastole (LASc and LASp, respectively) corresponds to conduit and pump function in the apical 4-chamber **(C)** and 2-chamber **(D)** views. The white dotted curved line indicates the average of LA strain. LASr, peak left atrial strain during ventricular systole; LASc, peak left atrial strain during early diastole; LASp, peak left atrial strain during atrial systole.

Strain curves were used to evaluate three LA phasic functions: reservoir, conduit, and pump function (Figure 2). LA longitudinal strain reflects reservoir function (LAS_r_), and LA pump function (LAS_p_) corresponds to LA strain at the onset of the P wave; LA conduit function (LAS_c_) is the difference between the two (i.e., LAS_r_ – LAS_p_) (6, 12). LV global longitudinal strain was calculated as the average negative peak of longitudinal strain in the apical 4-, 3-, and 2-chamber views, in accordance with current guidelines (13).

### Statistical Analysis

For continuous variables whose data were normally distributed, the Tukey-Kramer test was used; the data are presented as mean ± SD. In cases of non-normal distribution, the Kruskal–Wallis test was used and the data are presented as median (lower and upper limits of interquartile range). For categorical variables, the chi-square test or Fisher's exact test was applied to the data, as appropriate. Spearman's correlation analysis was used to investigate correlations, and linear regression analysis to investigate relationships between different variables. Multivariate regression analysis, using a forward stepwise algorithm, was used to investigate relations between CAVI value and other variables.

The SPSS software package (PASW Statistics 25, Chicago, IL, USA) was used for all statistical analyses. Statistical significance was set at *P* < 0.05.

## Results

### Patient Characteristics

Data from 165 patients were used. Table 1 shows their clinical characteristics. Patients were stratified into 3 groups (group L, *n* = 34; group M, *n* = 94; group H, *n* = 37) according to CAVI value relative to mean CAVI value for age. There were no significant differences between the groups in terms of age, sex, or systolic blood pressure.

**Table 1:** Comparison of the clinical characteristics of the study subjects among the study groups.

**Characteristics**	**Total (*n* = 165)**	**Group L (*n* = 34)**	**Group M (*n* = 94)**	**Group H (*n* = 37)**	***P-* value**
Age (years)	66.5 ± 11.7	68.3 ± 13.2	67.3 ± 10.5	66.1 ± 12.2	0.418
Male, *n* (%)	119 (72)	23 (62)	73 (78)	23 (67)	0.165
Body mass index (kg/m^2^)	23.9 ± 3.8	23.3 ± 4.5	23.6 ± 3.4	25 ± 4.5	0.124
Heart rate (beat/min)	69 ± 14	69 ± 13	69 ± 14	71 ± 14	0.756
Systolic blood pressure (mmHg)	135 ± 18	130 ± 11	134 ± 20	138 ± 20	0.215
Diastolic blood pressure (mmHg)	82 ± 12	78 ± 12	82 ± 11	89 ± 17	0.275
Diabetes, *n* (%)	65 (39)	8 (20)	34 (40)	23 (60)	<0.01
Hyperlipidemia, *n* (%)	102 (62)	14 (40)	59 (60)	29 (80)	<0.01
Creatinine (mg/dL)	0.89 ± 0.8	1.17 ± 1.69	0.84 ± 0.28	0.77 ± 0.26	0.12
HbA1c (%)	6.9 ± 2.1	6.5 ± 1.8	6.8 ± 2.1	7.2 ± 1.9	0.213
B-type natriuretic peptide (pg/mL)	31.2 ± 26.9	27.8 ± 21	31.3 ± 35.8	41.22 ± 55.8	0.193
CAVI	8.9 (8.2, 9.7)	7.7 (7.1, 8.2)	9.0 (8.4, 9.5)^*^	10.2 (9.2, 10.5)^*^, ^**^	<0.0001
**Medications**					
Diuretics, *n* (%)	11(6)	1(3)	9(10)	1(3)	0.391
α-blockers, *n* (%)	2(1)	0(0)	1(1)	1(3)	0.571
β-blockers, *n* (%)	24(15)	7(19)	10(11)	7(21)	0.256
ACEI/ARB, *n* (%)	50(30)	9(24)	27(29)	14(41)	0.511
Calcium blockers, *n* (%)	67(41)	17(50)	36(38)	14(38)	0.456

*Data are presented as mean ± standard deviation (SD) or median (interquartile range). CAVI, cardio-ankle vascular index; ACEI, angiotensin-converting enzyme inhibitor; ARB, angiotensin II receptor blocker*.

* *P < 0.0001 compared with Group L*,

** *P < 0.0001 compared with Group M*.

The patients' echocardiographic variables are summarized in Table 2. Regarding the results for two-dimensional echocardiography, patients in group H had significantly higher LV mass index (*P* < 0.001) and lower E wave, *E*/*A* ratio, and *e*′ (*P* < 0.05 for all three). In contrast, no significant intergroup differences were found for *E*/*e*' ratio or LA volume index. LVEF also showed no significant intergroup differences.

**Table 2:** Comparison of echocardiographic parameters among the study groups.

**Parameter**	**Total (*n* = 165)**	**Group L (*n* = 34)**	**Group M (*n* = 94)**	**Group H (*n* = 37)**	***P*-value**
**Two-dimensional echocardiography**					
LV end-diastolic diameter (mm)	41.9 (39.2, 45,0)	42.1 (38.0, 47.5)	42.2 (39.1, 45.0)	42.6 (39.8, 45.3)	0.128
LV end-systolic diameter (mm)	25.4 ± 4.0	26.3 ± 4.8	25.8 ± 3.3	26 ± 4.6	0.216
LV ejection fraction (%)	67.7 ± 4.6	68.3 ± 4.7	68 ± 4.1	67.2 ± 5.7	0.595
LV mass index (g/m^2^)	76.4 ± 25.4	75.4 ± 28.4	71.2 ± 23.5	90.5 ± 25.7^**^, ^***^	<0.001
E wave (cm/s)	61.9 ± 16.7	66 ± 16.2	62.5 ± 17	56.3 ± 15.4^**^	<0.05
A wave (cm/s)	79.1 ± 22.8	83.5 ± 27.6	76.3 ± 20.4	82.1 ± 23.1	0.201
E/A ratio	0.85 ± 0.42	0.95 ± 0.67	0.86 ± 0.33	0.71 ± 0.24^**^	<0.05
e' (cm/s)	5.8 ± 1.9	6.3 ± 2.3	5.9 ± 1.7	5.2 ± 1.7^**^	<0.05
E/e' ratio	11.4 ± 3.9	11.5 ± 3.9	11.3 ± 4.0	11.5 ± 3.7	0.951
LA volume index (ml/m^2^)	28.4 ± 9.4	26.1 ± 7.6	29.0 ± 9.3	30.0 ± 10.5	0.49
**Speckle-tracking echocardiography**					
LA reservoir strain (%)	22.5 ± 7.4	27.8 ± 9.3	21.8 ± 6.5^*^	19.4 ± 4.7^*^	<0.001
LA conduit strain (%)	10.7 ± 5.4	15.3 ± 7.5	10.2 ± 3.8^*^	7.5 ± 3.3^*, ^***^^	<0.001
LA pump strain (%)	11.9 (9.4, 14.0)	12.0 (9.5, 15.6)	11.9 (8.7, 13.5)	11.4 (9.8, 14.0)	0.493
LV global longitudinal strain (%)	−19.2(−21.1, −16.9)	−19.5 (−22.1, −17.8)	−19.0(−21.1, −17.3)	−18.0(−21.0, −14.1) ^**^	<0.05

*Data are presented as mean ± standard deviation (SD) or median (interquartile range). E, peak early diastolic velocity of transmitral flow; A, peak atrial systolic velocity of transmitral flow; e', peak early diastolic mitral annular motion velocity; LV, left ventricular; LA, left atrium*.

* *P < 0.0001 compared with Group L*,

** *P < 0.05 compared with Group L*,

*** *P < 0.001 compared with Group M*.

Regarding the results for speckle-tracking echocardiography, values for LA reservoir strain and LA conduit strain were significantly higher in group L than in group M and group H (*P* < 0.0001 for both comparisons), with the greatest difference between groups L and H. Although LVEF did not differ significantly among the 3 groups, when compared to group L, group H demonstrated a significantly lower LV global longitudinal strain (*P* < 0.05).

### Association of CAVI Value With Clinical Characteristics and Echocardiographic Variables

**Figure 3:**
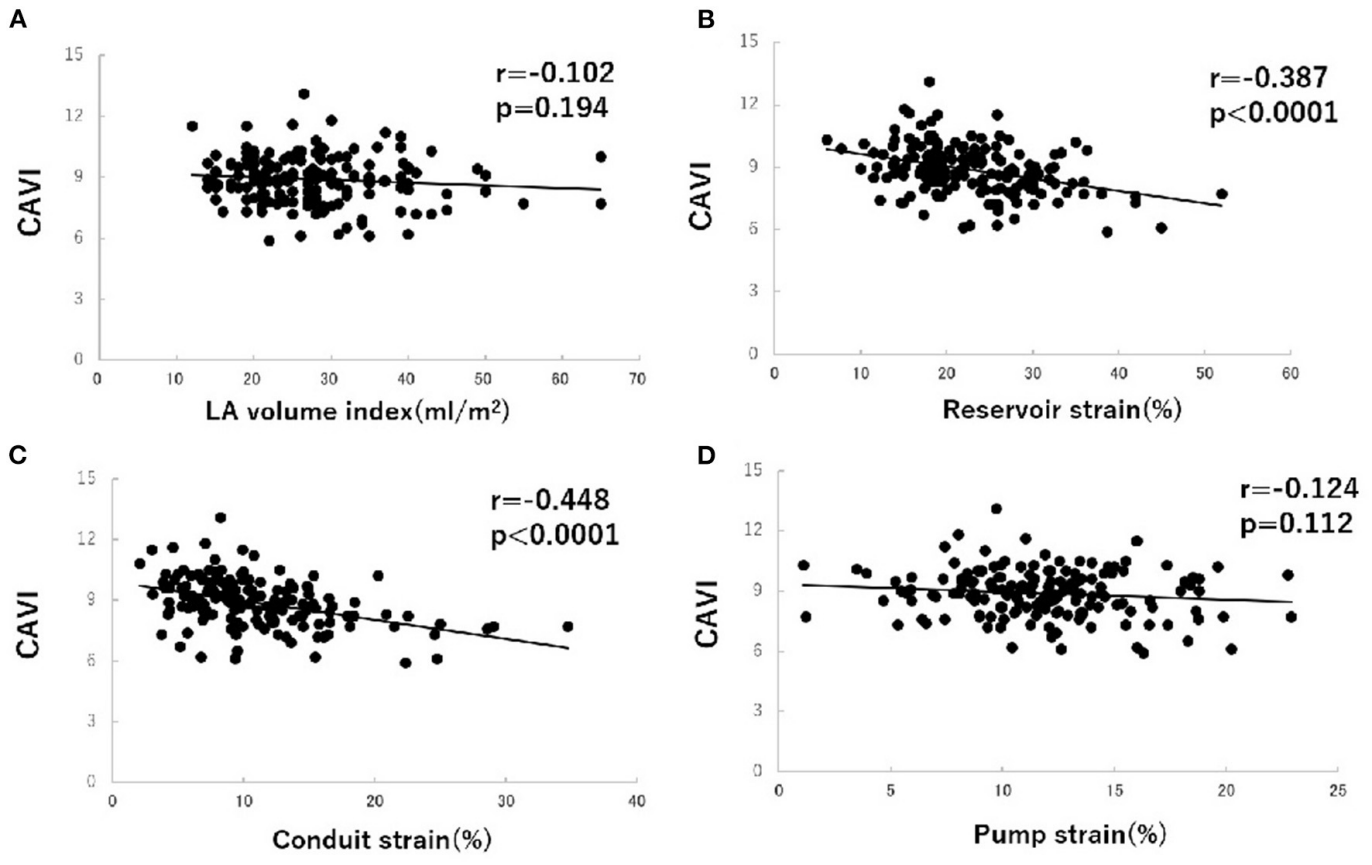
Correlations between cardio-ankle vascular index (CAVI) value and left atrial (LA) volume index **(A)**, LA phasic function **(B–D)** in hypertensive patients with preserved ejection fraction. CAVI, cardio-ankle vascular index; LA, left atrium.

To investigate factors affecting CAVI value, univariate and multivariate analyses were carried out using data for the patients' clinical characteristics and echocardiographic variables. The results of univariate analysis showed significant correlations between CAVI and age (*r* = 0.338, *P* < 0.0001), systolic blood pressure (*r* = 0.208, *P* = 0.007), E wave (*r* = −0.206, *P* = 0.008), *E*/*A* ratio (*r* = −0.265, *P* = 0.001), *e*' (*r* = −0.345, *P* < 0.0001), LA reservoir strain (*r* = −0.387, *P* < 0.0001), and LA conduit strain (*r* = −0.448, *P* < 0.0001) (Table 3 and Figure 3). Of these characteristics and variables, the results of multiple linear regression analysis showed CAVI to be independently related to age (β = 0.241, *P* = 0.002) and LA conduit strain (β = −0.386, *P* = 0.021) (Table 3).

**Table 3:** Univariate and multivariate analyses of the association of cardio-ankle vascular index with clinical variables and echocardiographic parameters.

**Total (*n* = 165)**	**Univariate**	**Multivariate**
	***r***	***P*-value**	**β**	***P*-value**
Age (years)	0.338	<0.0001	0.241	0.002
Sex (male = 1, female = 0)	−0.049	0.53		
Body mass index (kg/m^2^)	−0.022	0.78		
Heart rate (beat/min)	−0.024	0.755		
Systolic blood pressure (mmHg)	0.208	0.007	0.082	0.258
Diastolic blood pressure (mmHg)	0.042	0.596		
Diabetes	0.261	0.061		
Hyperlipidemia	0.136	0.082		
**Two-dimensional echocardiography**				
LV end-diastolic diameter (mm)	−0.108	0.168		
LV end-systolic diameter (mm)	−0.037	0.635		
LV ejection fraction (%)	0.012	0.883		
LV mass index (g/m^2^)	0.075	0.338		
E wave (cm/s)	−0.206	0.008	−0.068	0.394
A wave (cm/s)	0.138	0.077		
E/A ratio	−0.265	0.001	0.025	0.778
e' (cm/s)	−0.345	<0.0001	−0.092	0.263
E/e' ratio	0.095	0.227		
LA volume index (ml/m^2^)	−0.102	0.194		
**Speckle-tracking echocardiography**				
LA reservoir strain (%)	−0.387	<0.0001	0.055	0.721
LA conduit strain (%)	−0.448	<0.0001	−0.386	0.021
LA pump strain (%)	−0.124	0.112		
LV global longitudinal strain (%)	0.115	0.143		

*β, standardized regression coefficient. E, peak early diastolic velocity of transmitral flow; A, peak atrial systolic velocity of transmitral flow; e', peak early diastolic mitral annular motion velocity; LV, left ventricular; LA, left atrium*.

## Discussion

LA function has three elements, based on the three phases of the cardiac cycle: reservoir, conduit, and pump function (14). The first and second of these functions are regulated by inflow into the pulmonary vein during early ventricular systole and early ventricular diastole, respectively, and the third by the atrial contraction that occurs during late ventricular diastole to enhance ventricular filling (15). In the early stages of hypertension, LA pump function may increase to compensate for impairment of LV diastolic function; it has been shown to be higher in patients with mild hypertension compared with control subjects, whereas reservoir and conduit function were unchanged (16, 17).

Arterial stiffness reduces the compliance of systemic arteries, and thus contributes to LV afterload (18). In patients with hypertension, stiffness of the carotid artery has been shown to be associated with LA structural remodeling (19). This finding supports the hypothesis of arterial–LA coupling in hypertension, and reduced systemic arterial compliance may be expected to impair LA phasic function.

The results of the present study are the first to show that increased arterial stiffness is related to impaired LA reservoir and conduit function even before the development of LA dilatation and LV hypertrophy. Furthermore, they show that LV filling pressure is not increased in hypertensive patients with preserved LVEF. These findings suggest that increased arterial stiffness may detect an overload in the early stages of interactions between cardiovascular abnormalities in hypertensive patients.

Arterial stiffness is independently associated with LA diameter in patients with hypertension (20) and obstructive sleep apnea (21). However, arterial–LA coupling is not fully understood. We found increased CAVI value to be independently associated with impaired LA phasic function but not LA volume index, which suggests that arterial stiffness may first manifest as impairment of LA conduit function, and that LA dilatation develops later as arterial stiffness increases. Therefore, we suggest that an abnormality in LA phasic function, related to increased arterial stiffness, may be an early indicator of LA remodeling, and that detection of LA strain may facilitate earlier detection of LA dilatation as a late marker of LA remodeling.

The primary mechanism by which LA dysfunction develops has been thought to be increased LA afterload due to impaired LV relaxation and increased filling pressure (22, 23). However, the increase in LV filling pressure may be insufficient to explain the LA dysfunction, and LA myocardial fibrosis may contribute to LA systolic and diastolic dysfunction (24). Our finding that LA conduit function is impaired before any increase in LV filling pressure in hypertensive patients with abnormal LV relaxation (*E*/*A* ratio < 1) suggests that LA stiffness is increased even when LV filling pressure is unchanged. Previously, impaired LV relaxation has been reported to be associated with decreased LA conduit function (25, 26).

We found that LV diastolic dysfunction due to mild hypertension with increased arterial stiffness initially results in decreased LA conduit function. This is followed by a decrease in LA reservoir function as LA dilatation and LV hypertrophy progress. Further LA dilatation likely reduces LA pump function, thus producing a substrate for atrial fibrillation. In cases of heart failure with preserved LVEF, in which increased LV filling pressure causes mild LV diastolic dysfunction, LA conduit function is the first LA phasic function to be impaired (27). Therefore, CAVI determination (alongside the use of other indices of arterial stiffness) and assessment of LA phasic function may be useful in detecting early-stage heart failure in hypertensive patients with preserved LVEF.

The present study had some limitations. First, most of the patients used antihypertensive agents. Second, our findings are based on limited data; they require confirmation by the results of large-scale multicenter studies. Third, no data from simultaneous invasive evaluation of LA and LV function were available, because our patients had preclinical, asymptomatic hypertension. Forth, the present study was a retrospective observational study. We believe that prospective observational studies are necessary in the future. Finally, we demonstrated the concept of arterial-LA coupling based on the data of correlations between CAVI and LA function in mild hypertensive patients. To make this relationship about cardio-vascular interaction clearer, further studies are needed for advanced hypertensive patients.

## Conclusion

In hypertensive patients with preserved LVEF, increased CAVI was shown to be associated with impaired LA phasic function, particularly LA conduit function, before the onset of LA and LV remodeling. Arterial stiffness, assessed by CAVI determination, may play a key role in the early stages of interactions between cardiovascular abnormalities in hypertensive patients. Health care management using CAVI might contribute to the delineation of asymptomatic pre heart failure patients in the early stage. Further this study is needed prospective large scale to determine whether CAVI may be a good tool to identify patients at risk for severe disease in heart failure preserved LVEF.

## Data Availability Statement

The original contributions presented in the study are included in the article/supplementary material, further inquiries can be directed to the corresponding author/s.

## Ethics Statement

The studies involving human participants were reviewed and approved by the study was approved by the Ethics Committee of Toho University Sakura Medical Center (S18097). Informed consent was obtained using the opt-out form on the website. The patients/participants provided their written informed consent to participate in this study.

## Author Contributions

TT and KS: study conception and writing. TT, KS, YM, and NTan: data collection and analysis. TT, KS, MI, and HK: investigation. All authors contributed to manuscript revision, read, and approved the submitted version.

## Conflict of Interest

The authors declare that the research was conducted in the absence of any commercial or financial relationships that could be construed as a potential conflict of interest.

## Publisher's Note

All claims expressed in this article are solely those of the authors and do not necessarily represent those of their affiliated organizations, or those of the publisher, the editors and the reviewers. Any product that may be evaluated in this article, or claim that may be made by its manufacturer, is not guaranteed or endorsed by the publisher.

## References

[1] Matsuda M Matsuda Y. Mechanism of left atrial enlargement related to ventricular diastolic impairment in hypertension. Clin Cardiol. (1996) 19:954–9. 10.1002/clc.4960191211 8957600

[2] Shirai K Utino J Otsuka K Tanaka M. A noble blood pressure-independent arterial wall stiffness parameter; cardio-ankle vascular index (CAVI). J Atheroscler Thromb. (2006) 13:101–7. 10.5551/jat.13.101 16733298

[3] Hayashi K Yamamoto T Takahara A Shirai K. Clinical assessment of arterial stiffness with cardio-ankle vascular index: theory and applications. J Hypertens. (2015) 33:1742–57. 10.1097/HJH.0000000000000651 26114836

[4] Abhayaratna WP Fatema K Barnes ME Seward JB Gersh BJ Bailcy KR. Left atrial reservoir function as a potent marker for first atrial fibrillation or flutter in persons > or = 65 years of age. Am J Cardiol. (2008) 101:1626–9. 10.1016/j.amjcard.2008.01.051 18489941

[5] Mondillo S Cameli M Caputo ML Lisi M Palmerini E Padeletti M. Early detection of left atrial strain abnormalities by speckle-tracking in hypertensive and diabetic patients with normal left atrial size. J Am Soc Echocardiogr. (2011) 24:898–908. 10.1016/j.echo.2011.04.014 21665431

[6] Yoshida Y Nakanishi K Daimon M Ishikawa J Sawada N Hirokawa M. Association of arterial stiffness with left atrial structure and phasic function: a community-based cohort study. J Hypertens. (2020) 38:1140–8. 10.1097/HJH.0000000000002367 32371804

[7] Namekata T Suzuki K Ishizuka N Shirai K. Establishing baseline criteria of cardio-ankle vascular index as a new indicator of arteriosclerosis: a cross-sectional study. BMC Cardiovasc Disord. (2011) 11:51. 10.1186/1471-2261-11-51 21831311 PMC3166915

[8] Lang RM Badano LP Mor-Avi V Afilalo J Armstrong A Ernande L. Recommendations for cardiac chamber quantification by echocardiography in adults: an update from the American society of Echocardiography and the European Association of Cardiovascular Imaging. J Am Soc Echocardiogr. (2015) 28:1–39. 10.1016/j.echo.2014.10.003 25559473

[9] Devereux RB Reicheck N. Echocardiographic determination of left ventricular mass in man. Anatomic validation of the method. Circulation. (1977) 55:613–8. 10.1161/01.CIR.55.4.613 138494

[10] Nagueh SF Smiseth OA Appleton CP Byrd BF III Dokainish H Edvardsen T. Recommendations for the evaluation of left ventricular diastolic function by echocardiography: an update from the American Society of Echocardiography and the European Association of Cardiovascular Imaging. J Am Soc Echocardiogr. (2016) 29:277–314. 10.1016/j.echo.2016.01.011 27037982

[11] Badano LP Kolias TJ Muraru D Abraham TP Aurigemma G Edvardsen T. Standardization of left atrial, right ventricular, and right atrial deformation imaging using two-dimentional speckle tracking echocardiography: a consensus document of the EACVI/ASE/Industry Task Force to standardize deformation imaging. Eur Heart J Cardiovasc Imaging. (2018) 19:591–600. 10.1093/ehjci/jey042 29596561

[12] Sugimoto T Robinet S Dulgheru R Bernard A Ilardi F Contu L. Echocardiographic reference ranges for normal left atrial function parameters: results from the ECVI NORRE study. Eur Heart J Cardiovasc Imaging. (2018) 19:630–8. 10.1093/ehjci/jey018 29529180

[13] Voigt JU Pedrizzetti G Lysyansky P Marwick TH Houle H Baumann R. Definitions for a common standard for 2D speckle tracking echocardiography: consensus document of the EACVI/ASE/Industry Task Force to standardize deformation imaging. Eur Heart J Cardiovasc Imaging. (2015) 16:1–11. 10.1093/ehjci/jeu184 25525063

[14] Rocca M Lancellotti P Popescu BA Pierard LA. Left atrial function: pathophysiology, echocardiographic assessment, and clinical applications. Heart. (2011) 97:1982–9. 10.1136/heartjnl-2011-300069 22058287

[15] Hoit BD: Left atrial size and function: role in prognosis. J Am Coll Cardiol. (2014). 63:493–505. 10.1016/j.jacc.2013.10.055 24291276

[16] Eshoo S Ross DL Thomas L. Impact of mild hypertension on left atrial size and function. Circ Cardiovasc Imaging. (2009) 2:93–9. 10.1161/CIRCIMAGING.108.793190 19808574

[17] Boyd AC Eshoo S Richards DA Thomas L. Hypertension accelerates the ‘normal' aging process with a premature increase in left atrial volume. J Am Soc Hypertens. (2013) 7:149–56. 10.1016/j.jash.2012.12.008 23428410

[18] Briand M Dumesnil JG Kadem L Tongue AG Rieu R Garcia D. Reduced systemic arterial compliance impacts significantly on left ventricular afterload and function in aortic stenosis: implication for diagnosis and treatment. J Am Coll Cardiol. (2005) 46:291–8. 10.1016/j.jacc.2004.10.081 16022957

[19] Jaroch J Rzyczkowska B Bociaga Z Loboz-Rudnicka M Kruszynska E Rychard W. Arterial-atrial coupling in untreated hypertension. Blood Press. (2015) 24:72–8. 10.3109/08037051.2014.986929 25545339

[20] Lantelme P Laurent S Besnard C Bricca G Vincent M Legedz L. Arterial stiffness is associated with left atrial size in hypertensive patients. Arch Cardiovasc Dis. (2008) 101:35–40. 10.1016/S1875-2136(08)70253-5 18391871

[21] Drager LF Bortolotto LA Pedrosa RP Krieger EM Lorenzi-Filho G. Left atrial diameter is independently associated with arterial stiffness in patients with obstructive sleep apnea: potential implications for atrial fibrillation. Int J Cardiol. (2010) 144:257–9. 10.1016/j.ijcard.2009.01.018 19211166

[22] Evin M Redheuil A Soulat G Perdrix L Ashrafpoor G Grion A. Left atrial aging: a cardiac magnetic resonance feature-tracking study. Am J Physiol Heart Circ Physiol. (2016) 310:H542–9. 10.1152/ajpheart.00504.2015 26747498

[23] Habibi M Samiei S Venkatesh B Opdahl A Helle-Valle TM Zareian M. Cardiac magnetic resonance-measured left atrial volume and function and incident atrial fibrillation: results from MESA (Multi-Ethnic Study of Atherosclerosis). Circ Cardiovasc Imaging. (2016) 9:e004299. 10.1161/CIRCIMAGING.115.004299 27511974 PMC4985021

[24] Ohtani K Yutani C Nagata S Koretsune Y Hori M Kamada T. High prevalence of atrial fibrosis in patients with dilated cardiomyopathy. J Am Coll Cardiol. (1995) 25:1162–9. 10.1016/0735-1097(94)00529-Y 7897130

[25] Eshoo S Boyd AC Ross DL Marwick TH Thomas L. Strain rate evaluation of phasic atrial function in hypertension. Heart. (2009) 95:1184–91. 10.1136/hrt.2008.156208 19398436

[26] Prioli A Marino P Lanzoni L Zardini P. Increasing degrees of left ventricular filling impairment modulate left atrial function in humans. Am J Cardiol. (1998) 82:756–61. 10.1016/S0002-9149(98)00452-4 9761086

[27] von Roeder M Rommel K-P Kowallick JT Blazek S Besler C Flengler K. Influence of left atrial function on exercise capacity and left ventricular function in patients with heart failure and preserved ejection fraction. Circ Cardiovasc Imaging. (2017) 10:e005467. 10.1161/CIRCIMAGING.116.005467 28360259

